# Genomic Selection for Yield and Seed Composition Traits Within an Applied Soybean Breeding Program

**DOI:** 10.1534/g3.118.200917

**Published:** 2019-05-14

**Authors:** Benjamin B. Stewart-Brown, Qijian Song, Justin N. Vaughn, Zenglu Li

**Affiliations:** * Institute of Plant Breeding, Genetics and Genomics and Dep. of Crop and Soil Sci., University of Georgia, Athens, GA 30602; † Soybean Genomics and Improvement Lab, USDA-ARS, Beltsville, MD 20705; ‡ Genomics and Bioinformatics Research Unit, USDA-ARS, Center for Applied Genetic Technologies, Athens, GA 30602

**Keywords:** Genomic selection, RR-BLUP, Seed composition, Seed yield, Soybean, Genomic Prediction, GenPred, Shared Data Resources

## Abstract

Genomic selection (GS) has become viable for selection of quantitative traits for which marker-assisted selection has often proven less effective. The potential of GS for soybean was characterized using 483 elite breeding lines, genotyped with BARCSoySNP6K iSelect BeadChips. Cross validation was performed using RR-BLUP and predictive abilities (*r*_MP_) of 0.81, 0.71, and 0.26 for protein, oil, and yield, were achieved at the largest tested training set size. Minimal differences were observed when comparing different marker densities and there appeared to be inflation in *r*_MP_ due to population structure. For comparison purposes, two additional methods to predict breeding values for lines of four bi-parental populations within the GS dataset were tested. The first method predicted within each bi-parental population (WP method) and utilized a training set of full-sibs of the validation set. The second method utilized a training set of all remaining breeding lines except for full-sibs of the validation set to predict across populations (AP method). The AP method is more practical as the WP method would likely delay the breeding cycle and leverage smaller training sets. Averaging across populations for protein and oil content, *r*_MP_ for the AP method (0.55, 0.30) approached *r*_MP_ for the WP method (0.60, 0.52). Though comparable, *r*_MP_ for yield was low for both AP and WP methods (0.12, 0.13). Based on increases in *r*_MP_ as training sets increased and the effectiveness of WP *vs.* AP method, the AP method could potentially improve with larger training sets and increased relatedness between training and validation sets.

Quantitative traits have proven difficult to select for using marker-assisted selection (MAS) based on the fact that they are polygenic and loci responsible for variation in these traits often have small effects. Meuwissen *et al.* (2001) introduced the concept of genomic selection (GS) to take advantage of genotypic data to predict the performance of genotypes for complex traits. The main difference between MAS and GS, is that GS utilizes all markers across the genome to predict the performance of traits of interest, while MAS relies on a few markers to select specific QTL often associated with qualitative traits. Heffner *et al.* (2010) reported that GS provided threefold and twofold genetic gain per year compared to MAS for maize and winter wheat when costs were equivalent. With the advent of new genotyping platforms, such as single nucleotide polymorphism (SNP) beadchip arrays, Diversity array Technology (DArT), and genotyping-by-sequencing (GBS), high-throughput genotyping has made GS more affordable and efficient (Jain *et al.* 2017). The basic concept behind GS is that a set of breeding materials is used as a training set (TS). The TS is both genotyped and phenotyped for traits of interest in order to calculate marker effects which then predict performance of a test set that has been genotyped but not phenotyped. These phenotypic predictions are often referred to as genomic estimated breeding values (GEBVs). To evaluate the effectiveness of GS, a process referred to as cross-validation is often implemented. Cross-validation involves bisecting a set of lines which has been both genotyped and phenotyped into a TS and a validation set (VS). The TS is used to estimate marker effects to calculate GEBVs for the VS. The GEBVs are correlated with the observed phenotypic values of the VS and this determines predictive ability (*r*_MP_) (Combs and Bernardo 2013; Jacobson *et al.* 2014). The higher the correlation coefficient, the higher the predictive ability, and the more successful prediction is deemed to be. Prediction accuracy (*r*_MG_) is sometimes estimated as *r*_MP_ divided by the square root of heritability ($\sqrt{h^{2}}$) as a way to estimate success relative to phenotypic selection (Dekkers 2007). Studies have extensively explored GS across many crops but most extensively in maize (*Zea mays* L.) (Bernardo and Yu 2007; Lorenzana and Bernardo 2009; Albrecht *et al.* 2011; Guo *et al.* 2012; Riedelsheimer *et al.* 2013; Crossa *et al.* 2014; Jacobson *et al.* 2014, Lian *et al.* 2014) and wheat (*Triticum aestivum* L.) (de Los Campos *et al.* 2009; Heffner *et al.* 2011a; Heffner *et al.* 2011b; Poland *et al.* 2012; Crossa *et al.* 2014; Heslot *et al.* 2014; Rutkoski *et al.* 2015; Isidro *et al.* 2015). There are several factors that often influence the accuracy of GS. These factors include but are not limited to trait architecture and heritability, training set size and composition, marker density, and statistical model for estimation of marker effects (Jannink *et al.* 2010).

Soybean (*Glycine max* L. merr) accounted for 61% of the world’s oilseed production in 2016 (American Soybean Association 2018) and is a vital source of both protein meal for animal feed and vegetable oil for human consumption (Huth 1995). There have been several studies examining the potential for GS in soybean but relatively few compared to maize and wheat. Jarquín *et al.* (2014) was one of the first studies examining the potential for GS in soybean for seed yield prediction. They reported a prediction accuracy of 0.64 for seed yield across 301 experimental lines from the University of Nebraska-Lincoln soybean breeding program and found little improvement in accuracy when training set size (*N*_P_) exceeded 100 breeding lines. Predicted success when performing GS tends to be higher in studies reporting results with prediction accuracy *vs.* predictive ability, especially for lower heritability traits. The BARCSoySNP6K iSelect BeadChip was used to genotype a mixed population of 235 soybean cultivars by Ma *et al.* (2016) and potential for GS was examined for plant height and seed yield. They reported an increase in prediction accuracy of 4% for yield when using haplotype block-based markers (*r*_MG_ = 0.49) *vs.* random (*r*_MG_ = 0.48) or equidistant marker sampling (*r*_MG_ = 0.47). The potential to utilize GS has also been investigated within larger populations such as the SoyNAM population which is composed of over 5500 lines across 40 bi-parental populations (Xavier *et al.* 2016). Traits investigated were seed yield, days to maturity, plant height, pod number, node number, and pods per node. They detected minimal difference in accuracy across 14 statistical models (*r*_MG_ = 0.60 - 0.61) as well as minimal difference between genotyping densities of 4077 (*r*_MG_ = 0.60) *vs.* 1020 SNPs (*r*_MG_ = 0.61). They determined the most important factor for improving accuracy was to increase training set size as they examined *N*_P_’s from 250 (*r*_MG_ = 0.38) up to 4000 lines (*r*_MG_ = 0.75). They reported significant improvements in accuracy up to 2000 individuals. Thus far, there is no GS study in soybean with materials from later maturity groups. Soybean was second behind corn in terms of total acreage planted in 2017 (USDA-NASS 2017) and considering the importance of soybean on a national and global level, more studies are needed to characterize the potential for GS in soybean for complex traits.

The objective of this study was to characterize the ability to perform GS in later maturity groups within an applied soybean breeding program at the University of Georgia (UGA) and explore the effects of trait architecture and heritability, training set size and composition, and genotyping marker density on prediction of seed yield (yield). Protein and oil content (protein and oil) were also predicted as these traits are important from a breeder’s perspective because of the dependence on soybean as a protein source in animal feed and as a source of vegetable oil. Predictive ability of these two traits has yet to be investigated in a soybean GS study.

## Materials and Methods

### Plant Materials

The original GS dataset consisted of 14 distinct experimental sets which included 540 RILs from 26 pedigrees (Table 1). Set1-8 formed four bi-parental populations (Pop1-4) composed of 84 F_5:7_ RILs each. Two sets were stratified based on the maturity within each population. Pop1 was developed from a cross of AU02-3104 × G00-3213. AU02-3104 is a MG VII soybean line developed at Auburn University which was derived from a cross of ‘NC-Raleigh’ (PI 641156) × G92-1110 (Burton *et al.* 2006). G00-3213 is also a MG VII soybean line but was developed at UGA and was derived from a cross of ‘Boggs’ × ‘N7001’ (Boerma *et al.* 2000; Carter *et al.* 2003). Pop2, 3, and 4 were developed from crosses of G10PR-10 × G10PR-56389R2, G10PR-56248R2 × G10PR-56389R2, and G93-2225 × G09PR-54329R2, respectively. These parents are MG VII soybean lines developed at UGA. These four populations were advanced using a modified single-seed descent method (Brim 1966) and were within their initial year of replicated yield testing. Set9-14 consisted of 34 advanced F_5:8_ RILs each, from multiple pedigrees, which had undergone an additional round of breeding selection based on the first year of replicated yield testing. Set9-11 consisted of RILs from 12 separate pedigrees (Ped 1-12) as well as breeding selections from Pop1 and 4 and these RILs were stratified into equal sets based on early, middle, and late maturity. Set12-14 was divided similarly but consisted of RILs from 10 separate pedigrees and breeding selections from Pop2 and 3. These 540 breeding lines represented a large portion of the diversity in the UGA Soybean Breeding pipeline. Fifty-five breeding lines were present in two separate sets (Set1-8 and Set9-14) and phenotypic data for these lines remained for best linear unbiased predictor (BLUP) calculation, but these breeding lines were removed from Set9-14 in the GS dataset to avoid biasing results by cause of having the same genotypes in both the TS and VS during prediction. Two lines from Pop2 were removed from the dataset based on improper clustering according to a principle component analysis (PCA) using genotypic data and a total of 483 lines remained within the GS dataset for analysis (Table 1).

**Table 1:** Summary of genomic selection (GS) dataset

Set	Generation	# of pedigrees per set	# of breeding lines per set	# of pedigrees for GS	# of breeding lines for GS	Oil (Y/N)	Protein (Y/N)	Yield (Y/N)	Descriptor for GS
Set1-2	F_5:7_	1	84	1	84	Y	Y	Y	Pop1
Set3-4	F_5:7_	1	84	1	84	Y	Y	Y	Pop2
Set5-6	F_5:7_	1	84	1	82	Y	Y	Y	Pop3
Set7-8	F_5:7_	1	84	1	84	Y	Y	Y	Pop4
Set9-11	F_5:8_	14	102	12	82	N	N	Y	Ped1-12
Set12-14	F_5:8_	12	102	10	67	Y	Y	Y	Ped13-22
			540		483				

### Genotyping and Population Structure Analysis

Four hundred and eighty-five RILs were genotyped for the original prediction dataset. For each RIL, 20 seeds were planted in Styrofoam cups in a University of Georgia greenhouse facility. Once seedling were 3 weeks old, leaf tissue was harvested in 50-ml Falcon tubes, lyophilized, and ground into fine powder for DNA extraction. DNA was extracted using a modified CTAB (cetyl trim ethyl ammonium bromide) method (Keim *et al.* 1988). Genotyping was performed at Soybean Genomics and Improvement Lab at USDA-ARS, Beltsville, MD using BARCSoySNP6K iSelect BeadChips, returning 5403 SNPs (Song *et al.* 2013). Physical distances of SNPs were initially from the genome assembly version Glyma.Wm82.a1 (Gmax1.01) (Schmutz *et al.* 2010) and were then converted to version Glyma.Wm82.a2 (Gmax2.0). SNPs mapped in Gmax1.01 but not Gmax2.0, were excluded from the analysis (Song *et al.* 2016).

In addition to monomorphic SNPs, SNPs with > 10% heterozygous genotypes, > 80% missing data, or minor allele frequency (MAF) < 0.05 were removed. There were 2647 polymorphic SNPs remaining for GS. Various marker densities (*N*_M_) were investigated for their effect on *r*_MP_. The *N*_M_ categories tested were 1) all SNPs (2647 SNPs); 2) tag SNPs (yield: 1459 SNPs, protein and oil: 1435 SNPs); 3) half tag (yield: 748 SNPs, protein and oil content: 718 SNPs); 4) 4^th^ tag (yield: 374 SNPs, protein and oil: 359 SNPs); 5) 8^th^ tag (yield: 187 SNPs, protein and oil: 180 SNPs). Each tag SNP represents a genomic region with high linkage disequilibrium (LD). Depending on the trait, the number of tag SNPs varied as a result of variation in the composition of the GS dataset. Tag SNPs were determined using tagger in Haploview using pairwise tagging only and an r^2^ threshold set at 0.8 (de Bakker *et al.* 2005; Barrett *et al.* 2005). From the set of tag SNPs, every other marker was selected as half tag, every fourth marker as 4^th^ tag, and every eighth as 8^th^ tag. Population structure was examined using BARCSoySNP6K iSelect BeadChip data and the GAPIT R package (Lipka *et al.* 2012). PCA’s were plotted for visualization using TIBCO Spotfire 6.5.1 (2014).

### Phenotyping

For Pop1-4, 84 RILs from each population were divided into two equal sets of 42 based on maturity for yield trials, and two elite checks were included in each set. Yield trials were conducted in two locations in Georgia (Athens and Plains) for Set1-8 over 2 years. For Set1-2, yield evaluations were conducted in 2014 and 2016, while Set3-6 were evaluated in 2015 and 2016. Set7-8 were evaluated in 2014 but only Athens in 2016 due to lack of seed. Sets evaluated in 2014 or 2015 were replicated in two blocks per environment in a randomized complete block design (RCBD) and sets evaluated in 2016 were replicated three blocks per environment. In addition to select lines from the Pop1-4, breeding lines from Ped1-12 and Ped13-22 were allocated to Set9-11 and Set12-14 by maturity. Yield evaluations were performed in 2015 for Set9-11 and in 2016 for Set12-14. Each set consisted of 34 RILs and two elite checks which were replicated in three blocks per environment in an RCBD and were evaluated at three of four locations (Athens, Plains, and Tifton, GA or Florence, SC).

Set1-8 were planted in two-row plots, 4.9 m long and 76 cm apart. Plots were end trimmed to 3.7 m at R5 or R6 stage. Both rows were harvested for yield determination and adjusted to 13% moisture. For Georgia locations, Set9-14 were planted in the same way except in four-row plots and the plots at the Tifton location were not end-trimmed. For the Florence location, RILs were planted in four-row plots which were 6.1 m long and 76 cm apart. Plots were end-trimmed to 5.5 m at R5 or R6 stage. The middle two rows were harvested for yield determination and adjusted to 13% moisture.

Days to maturity was defined as the number of days from 1 September to maturity and was recorded on all blocks at the Athens location. Seed composition (protein and oil content) were measured from the same seed sources harvested for yield evaluation. Seed composition was not measured for Set 9-11 because of seed quality issues in 2015, resulting in no seed composition measurements obtained for any genotypes that year. For Set1-6 and 12-14, seed composition was measured from both Athens and Plains in 2016. Set1-2 also had seed composition measured from both locations in 2014. Seed composition was measured for Set 7-8 in 2014 and 2016 but only from Athens. Crude protein and oil were analyzed on a sample of ∼250 seeds from each plot using a DA 7250 NIR analyzer (Perten, Springfield, IL).

### BLUP and Heritability

BLUP values were calculated using the lme4 package (Bates *et al.* 2015) in R for each genotype and trait to account for variation resulting from environmental factors and maturity. Factors in the random model for yield included genotype, environment (a combination of year and location), genotype x environment interaction, set within environment, and days to maturity. Factors in the random models for protein and oil content included genotype, environment, genotype × environment interaction, and set within environment. To investigate the normality of BLUP values for each phenotypic trait, kernel density plots were created in R.

Heritability was estimated for each trait utilizing the rrBLUP package (Endelman 2011) implemented in R. An additive relationship matrix was created using the A.mat function. Utilizing the additive relationship matrix and phenotypic BLUP values for each genotype, genetic and error variances were estimated using the kin.blup function. Narrow-sense heritability was then estimated using the additive genetic ($\sigma^{2}{}_{a})$ and error variance ($\sigma^{2}{}_{e})$ outputs from kin.blup using the following equation: $h^{2}=\sigma^{2}{}_{a}/(\sigma^{2}{}_{a}+\sigma^{2}{}_{e})$ (Endelman and Jannink 2012). Utilizing the efficient mixed model association algorithm (Kang *et al.* 2008), restricted maximum log-likelihood was used to calculate variance components from a mixed model. The mixed model implemented is defined here as $y∼N(1\mu +ZGZ\prime \sigma^{2}{}_{a}+I\sigma^{2}{}_{e}$), where y is a vector of phenotypic values, 1 is a column vector of ones, μ is a fixed effect for the overall mean, Z is the genotypic incidence matrix, and G is the genomic relationship matrix. This notation for calculation of genomic heritability (de Los Campos *et al.* 2015) is also represented in Xavier *et al.* (2016).

### Genomic Prediction

The ridge regression best linear unbiased prediction (RR-BLUP) modeling methodology was utilized for GS using the rrBLUP package (Endelman 2011) implemented in R. Three different genomic prediction methods were investigated for three traits: yield, protein, and oil. In the first two methods, both TS and VS were established, and cross-validation was conducted by taking a random sample at various TS sizes and predicting GEBVs of genotypes in the VS. The third method was performed in the same manner, but with the TS and VS from separate pools. The correlation between GEBVs and the observed BLUP values was recorded. The procedure was replicated 100 times and *r*_MP_ was the average of these 100 replications.

#### Genomic prediction across entire genomic selection dataset (EGSD method):

**Figure 1:**
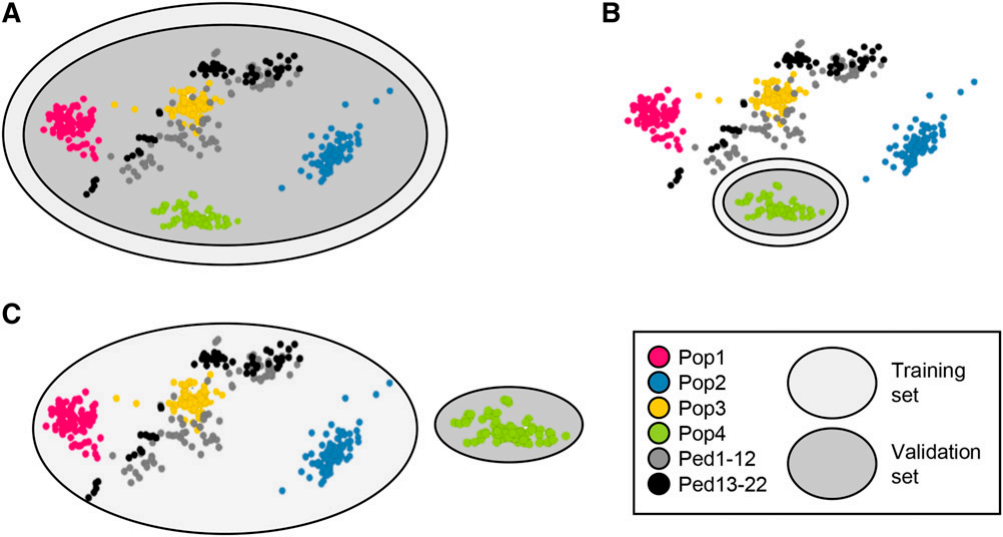
Diagram displaying the three methods performed for estimating predictive ability within the genomic selection dataset. (A) Perform cross-validation using the entire mixed population as both the validation set and training set (EGSD method), (B) Perform cross-validation within bi-parental populations using Pop1-4 individually as the validation set and training set (WP method); and (C) Predict across populations using one of Pop1-4 as the validation set and the remaining breeding lines as the training set (AP method).

The first prediction method examined the ability to predict when the TS and VS were pulled from the entire dataset at random. Predicting across mixed populations is a common approach for evaluating *r*_MP_ and provides a general idea of how well GS can function across all breeding materials (Jarquín *et al.* 2014; Xavier *et al.* 2016). For the EGSD method, *r*_MP_ for each trait was calculated across the entire dataset of 483 RILs for yield and 401 RILs for protein and oil (Figure 1A). The VS was composed of 50 randomly selected breeding lines from the entire dataset. The TS was composed of randomly selected breeding lines from the remaining genotypes at various TS sizes. Predictive ability was measured with marker density fixed at all SNPs at the following TS sizes: 50, 100, 150, 200, 250, 300, 350, and 400. For protein and oil, TS size was maximum at 350 as a result of a smaller subset of genotypes. Utilizing the maximum TS size for each trait, the effect of marker density on r_MP_ was investigated for various numbers of SNPs including all, tag, half tag, 4^th^ tag, and 8^th^ tag SNPs.

#### Genomic prediction within bi-parental RIL population (WP method):

Another goal of this study was to examine how well GS would function for predicting GEBVs within specifically each of the four bi-parental populations (Pop1-4), named as WP method. Both the TS and VS were the same bi-parental population and thus, full-sibs were used to predict full-sibs (Figure 1B). The GS dataset contained four bi-parental populations with 84 RILs each (Pop1-4). Cross-validation was performed similar to the EGSD method, except within Pop1-4 and a VS size of 20 RILs was used. Predictive ability was measured with marker density fixed at all SNPs at a TS size of 50 RILs. A limiting factor for the WP method is that TS size becomes restricted by the size of the bi-parental population. Another reason that this method may not be ideal is that the breeder would need replicated yield trials on a subset of a bi-parental population to generate phenotypic data and to train a model with which they can return to remnant seed of additional RILs to decide which plant rows to select for advancement (Jannink *et al.* 2010). For the purpose of this study, this strategy served mainly as a contrast to the third and most ideal GS method in which one of the four populations (Pop1-4) was the VS and all remaining breeding lines were compiled as the TS (AP method).

#### Genomic prediction across bi-parental RIL populations (AP method):

The AP method examined predictive ability when the VS was created from one of Pop1-4, but the TS was developed from the remaining breeding lines (Figure 1C). This method simulated a situation similar to how GS would actually be implemented in a breeding program in order to select better breeding lines within a newly developed population when no phenotypic data are available. Predictive ability was measured with marker density fixed at all SNPs and examined the following TS sizes: 50, 100, 150, 200, 250, 300, 350. For protein and oil, the largest TS size tested was 300, due to a smaller subset of genotypes having been phenotyped for these traits. Comparing WP and AP methods allows for an investigation of the ability to compensate for a decrease in genetic relatedness with an increase in TS size that can be achieved when using the AP method.

### Statistical Analysis

All significance tests of correlation were calculated using the Pearson’s product-moment correlation method via the ggpubr package (Kassambara 2017) in R. ANOVA was performed for each trait using the agricolae package (de Mendiburu 2017) to examine if there were statistical differences in *r*_MP_ resulting from changes in TS sizes and marker sets. For the ANOVA model, the dependent variable was predictive ability from each replication cycle and the independent variable was the factor of interest. A Fisher’s LSD multiple comparison test was performed to test differences of the means between different levels of each factor (α = 0.05).

### Data Availability

All data and code required to replicate the analyses are available in Files S1-5. File S1 contains the raw phenotypic data for calculation of BLUP values. File S2 contains the phenotypic BLUP values used for each method of GS. Worksheet 1 provides additional information for each genotype while Worksheets 2 and 3 contain information used for GS. Supplemental Data File S3 and S4 contains the genotypic data files used for prediction of seed yield and protein/oil content for each method of GS. Worksheet 1 of each file provides additional information for each genotype while Worksheets 1-10 contain genotypic data for the following marker densities: all SNPs, tag SNPs, half tag SNPs, 4^th^ tag SNPs, and 8^th^ tag SNPs. Odd sheets contain extra information while even sheets contain data used for GS. File S5 provides the r code used for calculation of predictive abilities which can be adapted to test all methods. Supplemental data files along with supplemental figures and tables are available at FigShare. R code used for calculation of predictive abilities is also available at FigShare. Supplemental material available at FigShare: https://doi.org/10.25387/g3.8121251.

## Results

### Population structure and genomic heritability

**Figure 2:**
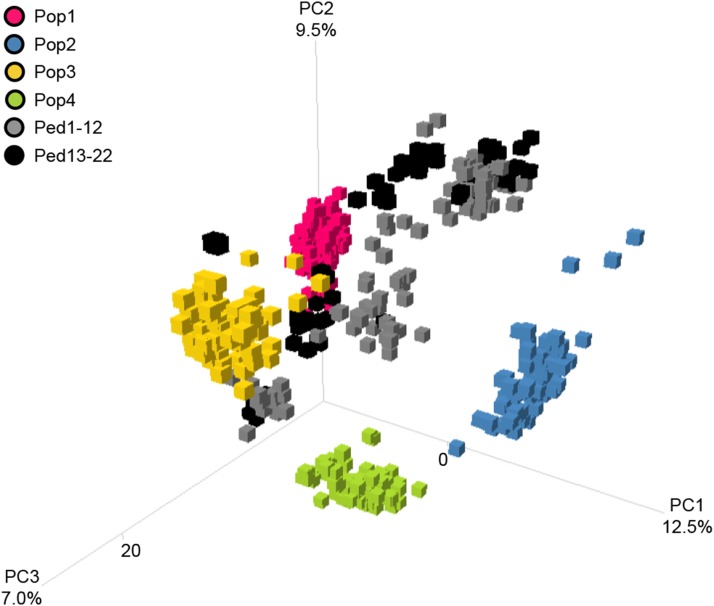
Principle component analysis of genomic selection dataset.

The GS dataset showed significant population structure due to the presence of four bi-parental RIL populations (Pop1-4) composing more than half of the entire dataset. The first, second, and third principal components explained 12.9, 9.5, and 7.0% of variation within the dataset, respectively (Figure 2). There was clear clustering within each of the four bi-parental populations and population structure among some of the advanced breeding lines from Ped5-22 was observed as several lines shared the same parentage. High genetic relatedness among many breeding lines was observed that led to clustering among several advanced breeding lines with Pop1-4.

BLUP values for each trait followed a normal distribution (Fig. S1). When RILs were separated by pedigree, it became evident that pedigrees varied in terms of their mean BLUP values for each trait (Fig. S2). When focusing on Pop1-4 which contributed to a majority of the GS dataset, there was evidence of populations which were numerically different in terms of protein and oil. Pop1 had higher oil and lower protein content compared to Pop2-4 in terms of mean BLUP values (Fig. S2B; Fig. S2C). Overall, protein and oil were significantly negatively correlated (r = −0.62; *P* < 1× 10^−15^). This supports prior reports of the inverse relationship between protein and oil content in soybean (Brummer *et al.* 1997; Brim and Burton 1979). Yield varied across pedigrees but the relative differences in yield between the four bi-parental populations compared to across all pedigrees was minimal (Fig. S2A). Though the correlations were not as strong, yield had a significant negative correlation with protein content (r = −0.10; *P* = 4.6 × 10^−2^) and had a significant positive correlation with oil content (r = 0.11; *P* = 3.4 × 10^−2^), which is consistent with previous reports (Chung *et al.* 2003).

BLUP values and an additive matrix of breeding material were used to compute genomic heritability for each trait via the kin.blup function in rrBLUP (Endelman 2011). Protein had the highest heritability with a genomic heritability of 0.82. Oil had a genomic heritability of 0.78 and yield had the lowest heritability trait at 0.17. Hwang *et al.* (2014) reported similarly high heritability estimates for protein and oil content and it is widely reported that yield is a low heritability trait for many crops, including soybean.

### Predictive ability across entire GS dataset (EGSD)

**Figure 3:**
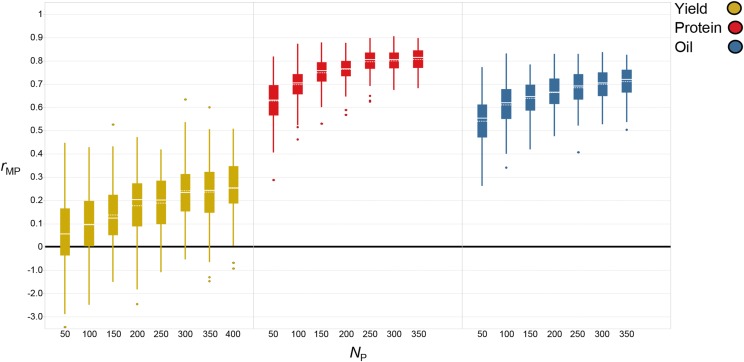
Boxplots of the effect of training set size (*N*_P_) on predictive ability (*r*_MP_) for each trait when utilizing the entire genomic selection dataset (EGSD) method. Solid line represents median and dotted line represents mean.

**Figure 4:**
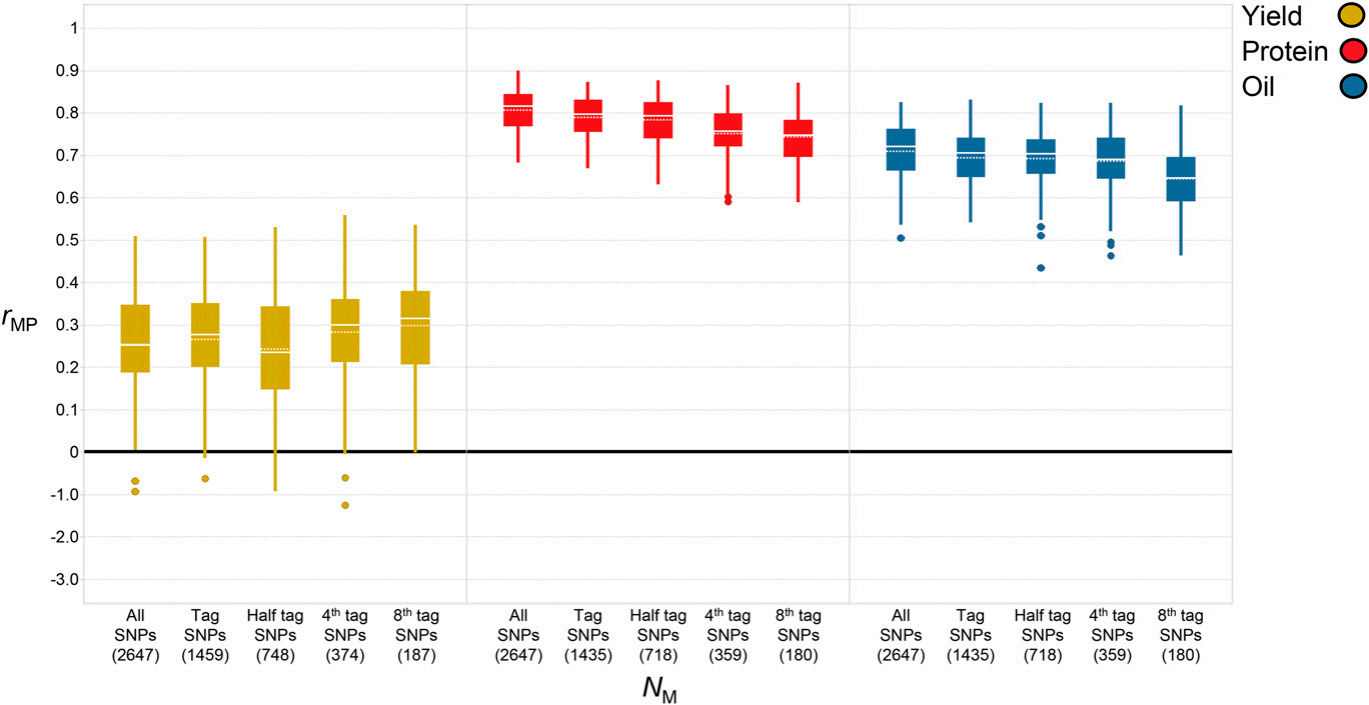
Boxplots of the effect of marker density (*N*_M_) on predictive ability (*r*_MP_) for each trait when utilizing the entire genomic selection dataset (EGSD) method. Number of markers indicated in parentheses. Solid line represents median and dotted line represents average.

Predictive ability for yield increased by 364% from 0.06 (*N*_P_ = 50) to 0.26 (*N*_P_ = 400) (Figure 3, percentages/significance tests were based on *r*_mp_ values in Table S1). As TS size increased by 50, predictive ability increased on average by 0.03. There were no significant differences in *r*_MP_ from a TS size of 300 (*r*_MP_ = 0.24) to 400 (*r*_MP_ = 0.26). Marker density appeared to have less impact on predictive ability compared to TS size. When comparing different marker densities, *r*_MP_ ranged from 0.30 (*N*_M_ = 8^th^ tag SNPs) to 0.24 (*N*_M_ = half tag SNPs) (Figure 4, percentages/significance tests were based on *r*_mp_ values in Table S2). Utilizing 8^th^ tag SNPs was only 0.04 greater in terms of *r*_MP_ compared to utilizing all SNPs so minor differences were present among marker densities.

For protein, TS size had a significant impact as well, evidenced by an increase in *r*_MP_ of 29% from 0.63 (*N*_P_ = 50) to 0.81 (*N*_P_ = 350) (Figure 3 and Table S1). The average increase in *r*_MP_ for each increase in TS size of 50 was 0.03, but gains were higher during the initial increase from 50 (*r*_MP_ = 0.63) to 100 (*r*_MP_ = 0.70). Predictive ability for protein began to diminish at larger TS sizes as *r*_MP_ only increased from 0.80 to 0.81 when TS size increased from 250 to 350. Marker density also had less impact on *r*_MP_ compared to TS size as predictive ability for protein decreased by only 8% from 0.81 (*N*_M_ = all SNPs) to 0.74 (*N*_M_ = 8^th^ tag SNPs) (Figure 4 and Table S2).

Oil was no exception to the trend of larger TS sizes resulting in higher predictive ability. Predictive ability increased by 31% from 0.54 (*N*_P_ = 50) to 0.71 (*N*_P_ = 350) (Figure 3 and Table S1). Similar to protein, the average gain in *r*_MP_ for each increase in TS size of 50 was 0.03, but the largest increase was observed from 50 (*r*_MP_ = 0.54) to 100 (*r*_MP_ = 0.61). Increases in *r*_MP_ were minimal as TS size increased from 250 (*r*_MP_ = 0.68) to 350 (*r*_MP_ = 0.71). As marker density decreased so did *r*_MP_ but the decrease was only 9% from 0.71 (*N*_M_ = all SNPs) to 0.64 (*N*_M_ = 8^th^ tag SNPs) (Figure 4 and Table S2).

There appeared to be a direct relationship between heritability and *r*_MP_ as the highest heritability traits (oil and protein) were more predictive than yield which had a lower heritability. By cause of the larger number of breeding lines which had been phenotyped for yield, a slightly larger TS size was tested compared to the other traits but when comparing traits at equal TS sizes, protein and oil were consistently higher than yield. For protein and oil, the highest predictive ability was achieved with all SNPs, while the highest predictive ability for yield was achieved with the lowest marker density of 8^th^ tag SNPs (Figure 4 and Table S2). Considering the SNP distribution decreased from ∼130 SNPs per chromosome (all SNPs) to ∼10 SNPs per chromosome (8^th^ tag SNPs), a more dramatic decrease in *r*_MP_ across all traits may have been anticipated. Overall, though statistical differences were present, it did not appear that decreasing marker density had a drastic effect on *r*_MP_ for any trait. On average, the difference in *r*_MP_ between the highest and lowest marker density across all traits was only 0.03.

### Predictive ability of individual bi-parental populations (WP *vs.* AP method)

#### Predictive ability averaged across populations (Pop1-4):

**Figure 5:**
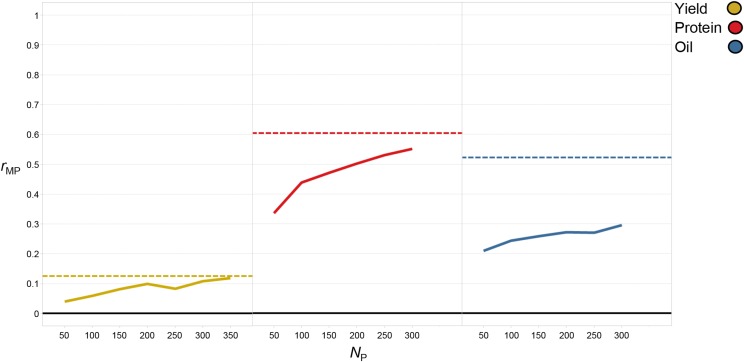
Graph displaying the effect of training set size (*N*_P_) on predictive ability (*r*_MP_) for each trait when contrasting the within population (WP) method *vs.* the across population (AP) method. *r*_MP_ was averaged across the four validation sets (Pop1-4). The WP method was indicated with a horizontal dashed line while the AP method was indicated with a solid trend line across TS sizes. For the WP method, a single training set size of 50 breeding lines was used.

The ability to predict lines within a bi-parental population using full-sib members of that population (WP method) *vs.* using the remaining breeding lines (AP method) was examined. For the initial analysis, r_MP_ was averaged across Pop1-4 for each trait at each TS size. Yield was the lowest heritability trait and achieved the lowest r_MP_ for WP (r_MP_ = 0.13) and AP (r_MP_ = 0.12) (Figure 5, percentages/significance tests were based on r_mp_ values in Table S3). Predictive ability for the AP method ranged from 0.04 (N_P_ = 50) to 0.12 (N_P_ =350). There were no statistical differences in predictive ability between a TS size of 300 or 350 for the AP method and a TS size of 50 for the WP method, as each achieved an r_mp_ of 0.13. When comparing both methods at an equal TS size (N_P_ = 50), predictive ability for the WP method was 205% higher than the AP method (0.13 *vs.* 0.04). Though this difference was significant, the WP method was still quite low in terms of predictive ability.

Protein was the highest heritability trait and achieved the highest *r*_MP_ for both WP (*r*_MP_ = 0.60) and AP methods (*r*_MP_ = 0.55). Predictive ability for the AP method ranged from 0.34 (*N*_P_ = 50) to 0.55 (*N*_P_ = 300) (Figure 5 and Table S3). For the AP method, the largest TS sizes of 250 and 300 were statistically equivalent in terms of *r*_MP_ (0.53 and 0.55). There was a 9% increase in *r*_MP_ when implementing the WP (*r*_MP_ = 0.60) *vs.* the AP method (*r*_MP_ = 0.55) at the maximum TS size (*N*_P_ = 300). When comparing both methods at an equal TS size (*N*_P_ = 50), predictive ability for the WP method was 80% higher than the AP method (0.60 *vs.* 0.34).

Oil was the second highest heritability trait and achieved the second highest *r*_MP_ for WP (*r*_MP_ = 0.52) and AP (*r*_MP_ = 0.30) (Figure 5 and Table S3). Predictive ability for the WP method was comparable to protein but when comparing values for the AP method was almost half. Predictive ability for the AP method ranged from 0.21 (*N*_P_ = 50) to 0.30 (*N*_P_ = 300) and TS sizes from 200 to 300 were statistically equivalent in terms of *r*_MP_ (0.27 to 0.30). There was an increase in *r*_MP_ of 76% when implementing the WP (*r*_MP_ = 0.52) *vs.* the AP method (*r*_MP_ = 0.30) at the maximum TS size (*N*_P_ = 300). When comparing both methods at an equal TS size (*N*_P_ = 50), predictive ability for the WP method was 149% higher than the AP method (0.52 *vs.* 0.21), comparable to protein. Both protein and oil had smaller increases in percentage compared to yield, but this was largely influenced by how low the predictive ability was for yield when utilizing the AP method.

For each trait, a higher or at least equivalent predictive ability was achievable when implementing WP *vs.* AP even though the maximum TS size achievable for AP was significantly larger (oil and protein: 50 *vs.* 300, yield: 50 *vs.* 350). When comparing both methods at an equal TS size of 50 (max *N*_P_ for WP), predictive ability was higher when implementing WP *vs.* AP for all traits, further highlighting the advantage of the WP *vs.* AP method.

#### Predictive ability of each individual bi-parental population (Pop1-4):

After investigating how the WP and AP methods compared on average across Pop1-4, individual populations were investigating to see if there were trends unique to any individual population. For yield, Pop1-4 achieved an *r*_MP_ of 0.04, 0.21, 0.25, and 0.01, respectively, when utilizing the WP method. (Fig. S3A, percentages/significance tests were based on *r*_mp_ values in Table S4). For the AP method, Pop1-4 achieved a maximum *r*_MP_ of 0.12 (*N*_P_ = 250 or 350), 0.10 (*N*_P_ = 350), 0.11 (*N*_P_ = 300), and 0.18 (NP = 350), respectively (Fig. S3A and Table S4). Predictive ability for yield was overall significantly lower than those for protein and oil in each population (Fig. S3A). As TS size increased for AP, *r*_MP_ tended to increase but fluctuated drastically throughout this trend. The WP method was significantly more effective in Pop2 and Pop3 compared to the AP method. For Pop1 and Pop4, the WP method performed poorly, and the highest prediction was achieved when implementing the AP method. Yield was far more population dependent compared to protein and oil in terms of prediction.

For protein, Pop1-4 achieved an *r*_MP_ of 0.64, 0.73, 0.61, and 0.43, respectively, when utilizing the WP method (Fig. S3B and Table S4). For the AP method, Pop1-4 achieved a maximum *r*_MP_ of 0.57 (*N*_P_ = 300), 0.55 (*N*_P_ = 300), 0.64 (*N*_P_ = 300), and 0.45 (*N*_P_ = 300), respectively (Fig. S3B and Table S4). As TS size increased, *r*_MP_ tended to increase when utilizing the AP method (Fig. S3B). For Pop1 and Pop2, *r*_MP_ for WP was significantly higher compared to AP when comparing the highest measured *r*_MP_ for each population. When comparing *r*_MP_ for Pop4, there was no significant difference between WP and AP at the largest tested TS size. Utilizing AP for Pop3, predictive ability of WP was surpassed starting at a TS size of 200. Though predictive ability for AP was higher, there were no significant differences when compared to WP at a TS size of 50.

For oil, Pop1-4 achieved an *r*_MP_ of 0.64, 0.36, 0.63, and 0.46, respectively, when utilizing the WP method (Fig. S3C and Table S4). When utilizing the AP method, Pop1-4 achieved a maximum *r*_MP_ of 0.12 (*N*_P_ = 50 or 200), 0.25 (*N*_P_ = 250 or 300), 0.48 (*N*_P_ = 250) and 0.36 (*N*_P_ = 300) (Fig. S3C and Table S4). Similar to protein, as TS size increased, *r*_MP_ tended to increase for AP (Fig. S3C). Even though the highest TS size did not always possess the highest *r*_MP_ in each individual population, it was statistically equivalent for each. WP was significantly more effective for prediction of Pop1-4 compared to AP. When comparing the highest *r*_MP_ for each population, Pop1 showed the largest discrepancy in ability to predict as AP was 18% of WP in terms of *r*_MP_. Predictive ability utilizing AP for Pop2-4 was on average 76% of *r*_MP_ utilizing WP. Also, Pop1 seemed to be the only population where predictive ability stagnated completely as there were no significant differences from 50 to 300 lines.

## Discussion

In previous literature, GS has shown potential to improve the rate of genetic gain over MAS for quantitative traits. Studies have been performed extensively in crops such as maize and wheat, but soybean has had comparably few studies investigating the potential for GS. Predictive ability for yield was targeted in this study as increasing yield is a primary focus of soybean breeders. The potential to perform GS for protein and oil was also investigated as it is important to increase protein and oil considering soybean is the main source of protein for animal feed and a major source of vegetable oil.

Three distinct methods of evaluating potential for GS were tested. The EGSD method was the most traditional approach in which the entire dataset was sampled for both the TS and VS. Two additional methods were then compared to examine how GS performed within bi-parental populations when genetic relationships were strongest, compared to a realistic scenario in which GEBVs were predicted for RILs within each bi-parental population using all other breeding lines as a training population. This last method demonstrates the most efficient way that GS could be implemented within a breeding program for plant row selection in order to make more informative decisions on which genotypes should be placed into advanced yield trials in cooperation with breeder notes.

### Predictive ability across entire GS dataset (EGSD method)

When performing cross-validation across the entire dataset, increasing TS size showed continuous increases in *r*_MP_ for all three traits of interest. Predictive abilities of 0.81, 0.71, and 0.26 for protein, oil, and yield, respectively, were achieved at the largest tested training set size. Jarquín *et al.* (2014) and Xavier *et al.* (2016) reported prediction accuracies of 0.64 and 0.75 for yield, which were calculated using *r*_MP_ divided by $\sqrt{h^{2}}$. Prediction accuracy, especially for lower heritability traits such as yield, is often much higher than predictive ability as a result of dividing by $\sqrt{h^{2}}$. This was observed in this study as prediction accuracies of 0.89, 0.80, and 0.63 were calculated for protein, oil, and yield, respectively, which is comparable with previous reports (Jarquín *et al.* 2014; Xavier *et al.* 2016). Though increases in *r*_MP_ continued as TS size increased, it appeared that gains for each trait diminished around 250 to 300 RILs. Jarquín *et al.* (2014) performed a cross-validation analysis in a mixed soybean population for yield and reported a similar result in that prediction accuracy increased as TS size increased, yet they witnessed a plateau in yield prediction around 100 breeding lines. Xavier *et al.* (2016) performed cross-validation across the entire SoyNAM population for yield and reported significant increases in prediction accuracy up to 2000 RILs. Different populations contain different levels of LD and substructure, so the ideal TS size for GS may be population dependent. Many studies have corroborated though that an increase in TS size will often result in an increase of *r*_MP_ with eventual diminishing returns (Lorenzana and Bernardo 2009; Guo *et al.* 2012; Heffner *et al.* 2011a; Heffner *et al.* 2011b; Jarquín *et al.* 2014; Xavier *et al.* 2016; Zhang *et al.* 2017). The improved ability to predict GEBVs as TS size increased is a reflection of the fact that there is an increased replication of alleles within a TS, allowing for a well-trained GS model. At smaller sizes, breeding lines with poor phenotypic data can negatively influence accurate estimations of allele effects. These outlier breeding lines are offset by increased replication as the TS size increases (Muir 2007). Also, as TS size increases, rare allele frequencies increase, which will help improve estimations of these marker effects (Jarquín *et al.* 2014).

Genomic heritability was calculated for each trait utilizing BLUP phenotypic values for each genotype and an additive kinship matrix via the kin.blup function (Endelman 2011). The narrow sense heritability estimates for protein, oil, and yield were 0.82, 0.78, and 0.17. It is not surprising that protein and oil content have higher heritability relative to yield based on the complex trait architecture and interactions both epistatically and environmentally that are often associated with yield. Heritability estimates for yield were low compared to previous GS studies investigating prediction potential for yield in soybean. Xavier *et al.* (2016) calculated heritability in a similar manner, estimating a heritability of 0.49 in 2013 and 0.41 in 2014 for yield. Since their heritability estimates were broken up by year, this eliminated the variance associated with genotype × year interactions.

Traits with higher heritability having higher *r*_MP_ values is a common occurrence in GS studies. Combs and Bernardo (2013) observed this trend with few exceptions when analyzing *r*_MP_ in maize, barley (*Hordeum vulgare* L.) and wheat populations. Heffner *et al.* (2011b) and Albrecht *et al.* (2011) also reported similar results. As there is often a strong relationship reported between heritability and *r*_MP_, increasing the heritability of the trait by improving phenotyping accuracy utilized in a GS model can be useful for better prediction. As a breeding program increases the size of the TS used for GS, one should investigate environments (location × year combinations) in which phenotypic traits have shown to have unusually low heritability as this may have been caused by odd environmental factors specific to that location within that year.

When holding TS size constant at the highest tested size for each trait, protein and oil content showed a decrease in *r*_MP_ as marker density decreased from all SNPs (2647 SNPs) to 8^th^ tag SNPs (180 SNPs), but the decrease in *r*_MP_ was only 0.07 for oil and 0.06 for protein. For yield, there were slight fluctuations in this trend and the highest *r*_MP_ was achieved with the lowest *N*_M_. Muir (2007) reported that increasing marker density can actually lead to a decrease in prediction accuracy in some situations and this is related to the increase in collinearity between markers (Whittaker *et al.* 2000). If TS sizes are not large enough, it is also possible that marker effects can be overestimated and this problem is confounded by the increased number of markers used for genotyping. Lorenzana and Bernardo (2009) demonstrated fluctuations in prediction accuracy related to marker density as they evaluated prediction of several agronomic traits within maize and barley populations. Within the maize population BM-TC1, they reported a higher accuracy at a marker density of 256 SNPs (*r*_MG_ = 0.56) compared to marker densities of 512 (*r*_MG_ = 0.55) and 768 SNPs (*r*_MG_ = 0.54). When assessing prediction of glucose concentration within the same population, they reported the highest accuracy achieved at a marker density of 512 SNPs (*r*_MG_ = 0.69), which was higher than the accuracy reported at the highest marker density of 1024 SNPs (*r*_MG_ = 0.67). Within a barley population derived from ‘Steptoe’ × ‘Morex’, a higher or equivalent accuracy was reported for grain yield and grain protein at 128 SNPs (*r*_MG_ = 0.62, 0.82) *vs.* the highest density at 223 SNPs (*r*_MG_ = 0.62). In this study, the difference between using all SNPs (2647) and 8^th^ tag SNPs (187 SNPs) was only an increase in *r*_MP_ of 0.04, even less than the difference for protein and oil. Several studies have reported that decreasing marker density can have minimal impacts on prediction (Lorenzana and Bernardo 2009; Lorenz *et al.* 2011; Heffner *et al.* 2011b). It is important that marker density is high enough to have linkage with QTL which may be responsible for variance in the quantitative trait of interest. Considering soybean has considerably high LD relative to other crops, it is not surprising that marker density seemed to have little effect on improving *r*_MP_.

**Figure 6:**
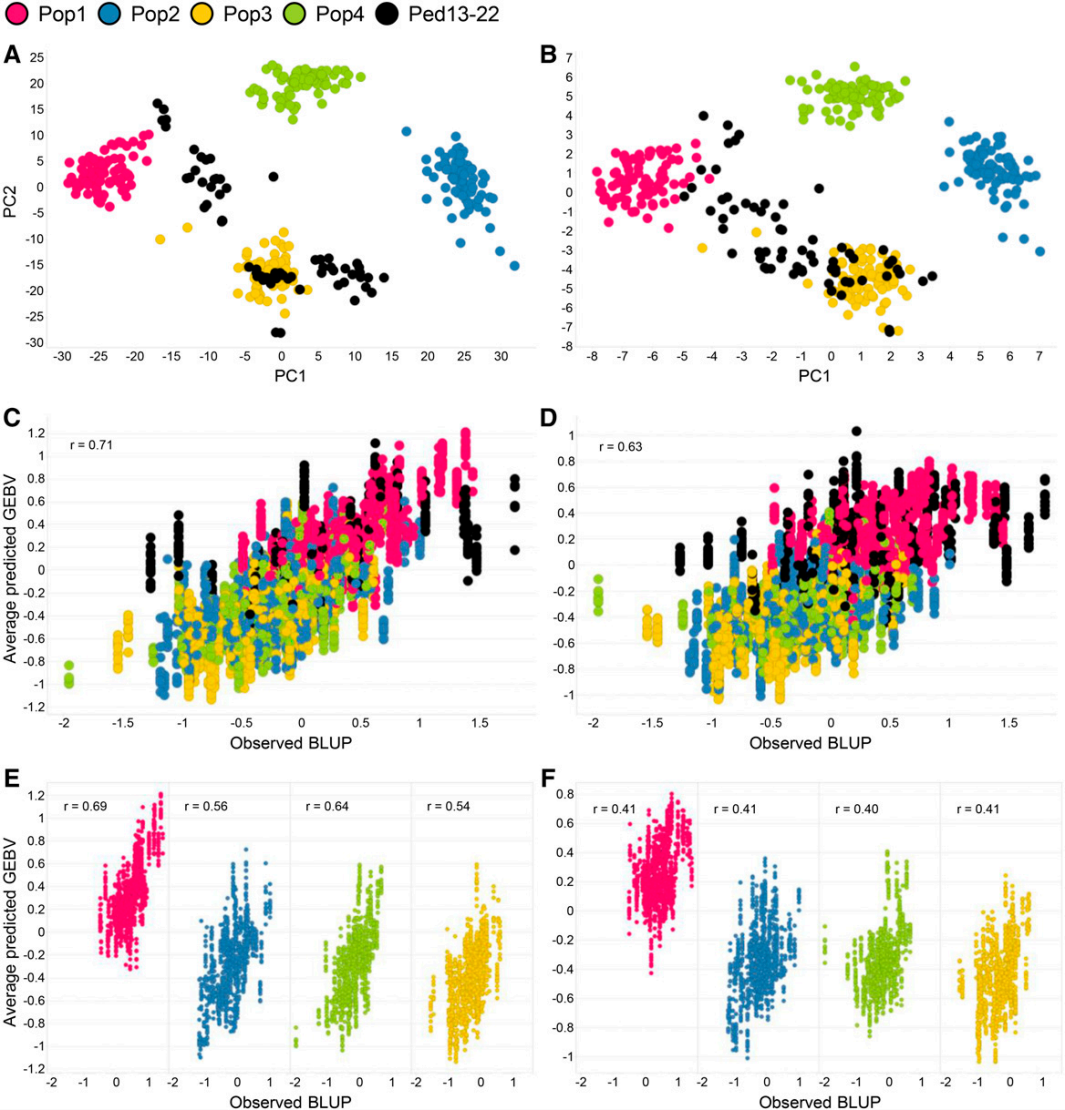
Effects of population structure on prediction of oil content when utilizing the entire genomic selection dataset (EGSD) method. (A) PCA of genomic prediction population using all SNPs. (B) PCA of genomic prediction population using 8^th^ tag SNPs. (C) Average predicted GEBV *vs.* observed BLUP values when using all SNPs. (D) Average predicted GEBV *vs.* observed BLUP values when using 8^th^ tag SNPs. (E) Average predicted GEBV *vs.* observed BLUP within Pop1-4 when using all SNPs. (F) Average predicted GEBV *vs.* observed BLUP within Pop1-4 when using 8^th^ tag SNPs. Correlation coefficients presented within scatterplots (C-F).

When examining the effects of different marker densities, there was a minimal change in *r*_MP_ even at the lowest marker density. It was hypothesized this may have partially been related to the strong population structure present within the GS dataset. The ability to differentiate bi-parental populations from each other *vs.* prediction within populations may be affecting predictive ability. Oil content was investigated to illustrate this concern. Population structure was first visualized via PCA at all SNPs compared to the lowest marker density, 8^th^ tag SNPs (Figure 6A and B). There was still identifiable population structure at the lowest marker density, indicating that an ability to differentiate each of the four bi-parental populations (Pop1-4) from each other at the lowest marker density remained. When examining the original oil BLUP values for each genotype, it was evident that Pop1 was higher in oil content compared to Pop2-4 (Fig. S2C). Thus, if there was an ability to genetically differentiate breeding lines from Pop1 compared to Pop2-4, these lines would be predicted to be higher in oil content compared to the other three populations. Pop1-4 influenced a large portion of *r*_MP_ because they composed ∼83% lines of the entire GS dataset for oil.

The average predicted oil GEBVs for each RIL were plotted against the observed oil BLUP values (Figure 6C and D). For all SNPs, the correlation coefficient between the average predicted oil GEBVs and observed oil BLUP values of the entire GS dataset was 0.71. For 8^th^ tag SNPs, the correlation between the observed and predicted values was 0.63 which was a 11% decrease. Within Pop1-4, decreases in correlation were 41% (0.69 to 0.41), 27% (0.56 to 0.41), 38% (0.64 to 0.40), and 24% (0.54 to 0.41), respectively (Figure 6E and F). Correlation coefficients decreased more within each individual population compared to across all populations, indicating that *r*_MP_ may be affected by differences between high and low oil populations as well as high and low oil breeding lines within these populations.

The EGSD approach is a common method to examine the potential for GS within a mixed population of breeding materials. It was observed that population structure can have a strong influence on *r*_MP_ and inflate confidence in prediction. Predicting across different populations also has the possibility of inflating *r*_MP_ due to the TS possibly containing full-sibs to genotypes placed in the VS unless precautions are taken to avoid this. Since breeders are often applying GS to make predictions in new unique parental combinations, having full-sibs in both the TS and VS is rare. Population structure can be accounted for by including population as an effect in a BLUP model. This would possibly mitigate an ability to identify that the worst line in one population may be better than the best line in another population. Caution should be used when assessing *r*_MP_ across different populations as one may be detecting more population differences than differences among the best and worst breeding lines within each population. Not only may this phenomenon be accounting for a lack of significant decreases in *r*_MP_ at extremely low marker densities, but it is most likely inflating *r*_MP_ at each level of marker density for the same reasons.

Predictive ability for yield was lower compared to protein and oil content by cause of the complexity and low heritability of yield but also partially because Pop1-4, which dominated the GS dataset, had similar mean yield BLUP values, so mean yield differences among these populations was not driving prediction as much as it appeared to be for protein and oil. Predictive ability is most likely inflated for many studies which combine multiple bi-parental populations in GS datasets, specifically when phenotypic means vary across populations. Previous literature has discussed the issues of population structure within GS but this usually refers to substructure within the VS, which is not properly represented within the TS and thus marker effects are not properly estimated (Guo *et al.* 2013; Crossa *et al.* 2014). Though this is an issue in GS, it is not the population structure related issue referred to here.

### Predictive ability of individual bi-parental populations (WP method *vs.* AP method)

The most common breeding pipeline for soybean begins with developing F_1_’s from unique parental combinations. The single seed descent method (SSD) advances lines until the F_4_ or F_5_ generation (Brim 1966). At this stage, hundreds of single plants are selected based on visual assessment of plants in the field. Selected single plants become plant rows which undergo another round of visual selection for key agronomic traits (*i.e.*, plant height, lodging, maturity) or plant row yield tests. Many plant rows across populations are often discarded based on breeder notes that can be heavily influenced by environmental factors including but not limited to soil conditions, field slope, mechanical damage, or disease/insect pressure. Single row measurements for traits such as yield, are time-consuming, labor-intensive, and often not reliable estimates (Sebastian *et al.* 2012). GS has the advantage of leveraging years and locations of replicated field trials to estimate marker effects in order to predict GEBVs for these plant rows that are ideally more reliable than simple visual assessments or single plot phenotyping. The advantage in utilizing GS at this stage *vs.* a visual assessment should warrant the cost, labor, and time associated with genotyping these plant rows if one is to effectively implement GS at this stage of their breeding program.

Two methods were compared for prediction of each individual bi-parental population (Pop1-4). For comparison purposes, maker density was fixed at all SNPs. Predictive ability was higher for higher heritability traits for both the WP and AP approaches. Utilizing the WP approach was often higher than prediction utilizing the AP approach. For WP, TS size was 50 and when averaging across Pop1-4, this was superior or at least statistically equivalent to a max TS size of 300 for protein and oil and 350 for yield. When comparing the two methodologies at the same TS size, the advantage of WP over AP was even more drastic for all traits. This was most likely resulting from the genetic relatedness between the TS and VS when using full-sibs via the WP approach. For WP, markers were in LD with QTL controlling variation for the traits of interest. Once unrelated materials were brought into the TS in the AP method, the loss in genetic relatedness between TS and VS resulted in a decrease in *r*_MP_ (Clark *et al.* 2012). This decrease in relatedness was most likely harming prediction as markers were in LD with QTL specific to populations in the TS and these QTL might not be represented in the VS (Lorenz *et al.* 2012). The strong subpopulation structure due to having large bi-parental populations in the TS exacerbated the issue as allele effects became increasingly biased toward the allele effects within these larger populations which were not represented in the VS (Guo *et al.* 2013; Crossa *et al.* 2014).

For higher heritability traits (*i.e.*, protein and oil), the AP method approached the WP method by taking advantage of larger TS sizes. This was likely due to the added replication of alleles allowing for more accurate estimates of allele effects. The high heritability of these traits implied that a large amount of variation controlled by genetics made marker effect estimates more accurate for prediction. Though this appeared to be the trend on average across populations, certain specific bi-parental populations could not approach predictive ability for the WP method, even at large TS sizes for high heritability traits. The AP approach for predicting oil content for Pop1 showed little success. This may have been a result of unique alleles specific to oil content being present in Pop1 yet largely absent from other breeding lines which composed the TS. This same trend did not occur for the other high heritability trait, protein, so it appeared to be specific to oil and not related to overall genetic relatedness between Pop1 and the other breeding lines.

Yield proved especially difficult to predict as prediction for both methods for each population was comparatively low. As TS size increased during implementation of the AP method, trends in *r*_MP_ varied far more for individual bi-parental populations for yield compared to protein and oil. There was some success using the WP approach for Pop2 and 3, but Pop1 and 4 did not predict well. Lian *et al.* (2014) predicted within 969 maize bi-parental populations and reported *r*_MP_ ranging from -0.34 to 0.89, providing evidence of the variability in predictive ability that can occur for yield even when predicting within populations. The AP methodology was largely unsuccessful for yield and seemed to only surpass WP in situations where *r*_MP_ was extremely low such as Pop1 and Pop4. It is possible the high level of structure within the training set may have attributed to this overall lack of success as allele effects were biased toward the bi-parental populations present within the TS. The complexity and low heritability of yield made variation in *r*_MP_ of different populations far greater compared to higher heritability traits such as protein and oil. Also, genotype × environment interactions were most likely harming prediction as alleles in one environment may have had opposing effects on yield in another for certain breeding lines.

There has been success predicting for various traits using approaches similar to the AP method but they have had the advantage of leveraging larger numbers of more related plant materials. Riedelsheimer *et al.* (2013) performed cross-validation within a mixed population of 635 maize doubled-haploid (DH) lines for several yield component traits. They reported that across all traits, a TS composed of DH lines which were full-sibs predicted significantly better than a TS composed of DH lines which were half-sibs and prediction was even worse if unrelated breeding lines were placed into the TS. They also reported having half-sibs present for both VS parents was significantly better than having half-sibs for one of the VS parents. Jacobson *et al.* (2014) developed a general combing ability (GCA) model in which maize inbreds were placed into a TS which were half-sibs with the VS and compared to pooling random inbreds in the TS. The GCA model significantly outperformed the random inbred model across 30 test populations for yield, moisture, and test weight. Jacobson *et al.* (2014) was able to leverage 970 testcross populations made available by Monsanto and although the approach was promising, this current study would have needed more extensive genotyping and phenotyping of material to have implemented a similar study in soybean. For the AP method, there were half-sibs present within the TS which may have led to some of the success in prediction, but not close to the numbers observed in these aforementioned studies.

Constructing GS models using full-sibs (WP method) appears to be effective for GS. This most likely delays the breeding cycle compared to leveraging previous phenotypes and genotypes to make predictions (AP method). Prediction of protein content showed the most promise for GS via the AP method as on average, the AP method predicted comparably to the WP method. For oil content, the same could largely be said, but one population decreased average AP predictive ability significantly, indicating that variability in prediction can occur depending on the population being predicted even for high heritably traits. Though successful prediction was achieved for protein and oil, the primary objective for soybean breeders is to make selections based on yield. It is assumed that the level of success achieved for yield within this study may not justify the cost and time needed to impose GS for yield. Though *r*_MP_ for yield was low compared to protein and oil, populations on average showed an upward trend and still made gains in r_MP_ at the highest TS size. Simply increasing TS size may not be the best solution as the literature has shown the benefits of increasing genetic relatedness between TS and VS. Studies in maize have reported success predicting across populations when leveraging half-sibs that represent both parents in the cross in addition to increasing TS size. Targeting this approach may be the best strategy for improving GS for yield in soybean in the future. A study investigating this has not yet been shown in soybean as many previous studies have evaluated prediction across mixed populations.

The success in prediction of protein and oil content alone may not warrant the application of GS as NIR spectrometry provides good estimates of these phenotypes with minimal time, labor, and cost. If predictive ability for yield could be increased enough to warrant genotyping of single-plant rows, acquiring predictions of protein and oil would be a logical additional step with minimal additional efforts.

### CONCLUSION

This study illustrated use of genomic selection for prediction of yield within a soybean breeding program. This was the first report indicating the success that can be achieved for higher heritability traits such as protein and oil content. Predictive ability can be inflated when there is population structure present in combination with differences in trait means across populations. Increased success across all traits can be attributed to increasing training set size more so than increased marker density, though benefits associated with training set size had eventual diminishing returns. Predictive ability can also be increased by building training sets with increased relatedness to validation sets. Yield was difficult to predict and this is most likely related to complex genotype × environment interactions, its highly quantitative nature, and a biasing of allele effects toward populations which dominated the training set. For future success, a larger training set size in combination with increased genetic relatedness between training and validation set could improve predictive ability in soybean as it has in maize.
